# Amelanotic Melanoma Treated as Fungal Infection for Years

**DOI:** 10.1155/2022/2598965

**Published:** 2022-11-07

**Authors:** Guilherme Kuceki, Dekker C. Deacon, Aaron M. Secrest

**Affiliations:** ^1^School of Medicine, University of Utah, Salt Lake, UT, USA; ^2^Department of Dermatology, University of Utah, Salt Lake, UT, USA; ^3^Huntsman Cancer Institute, University of Utah, Salt Lake, UT, USA; ^4^Department of Population Health Sciences, University of Utah, Salt Lake, UT, USA

## Abstract

This study describes a case of amelanotic lentigo maligna melanoma in a 69-year-old female that had been growing for approximately 5 years. The asymptomatic lesion had been previously diagnosed and treated as a fungal skin infection, an inflammatory rash, and an actinic keratosis that did not respond to standard treatments. Biopsy revealed confluent and nested atypical melanocytes at the dermal-epidermal junction, consistent with melanoma in situ. Excisional biopsy revealed invasive lentigo maligna melanoma, Breslow depth 0.3 mm, with positive melanoma in situ at margins. She is now 3 years post-Mohs surgery without recurrence. When working up a patient with a hypopigmented or inflammatory lesion not responding to standard therapies, physicians should always consider biopsy to rule out unusual neoplastic etiologies, such as amelanotic melanomas.

## 1. Introduction

Melanoma can occur on any skin or mucosal surface. Despite melanoma accounting for only 4% of all skin cancers, it is responsible for approximately half of skin cancer-related deaths [1, 2]. Amelanotic melanoma occurs in up to 1–8% of all melanomas; however, the actual percentage might be lower as many hypopigmented melanomas are characterized as amelanotic [3]. These lesions are difficult to diagnose due to their ability to masquerade as other hypopigmented lesions [4]. Herein, we describe an amelanotic melanoma that was treated as tinea corporis, nummular dermatitis, and actinic keratosis for 5 years before biopsy established the diagnosis and led to appropriate treatment.

## 2. Case Report

**Figure 1:**
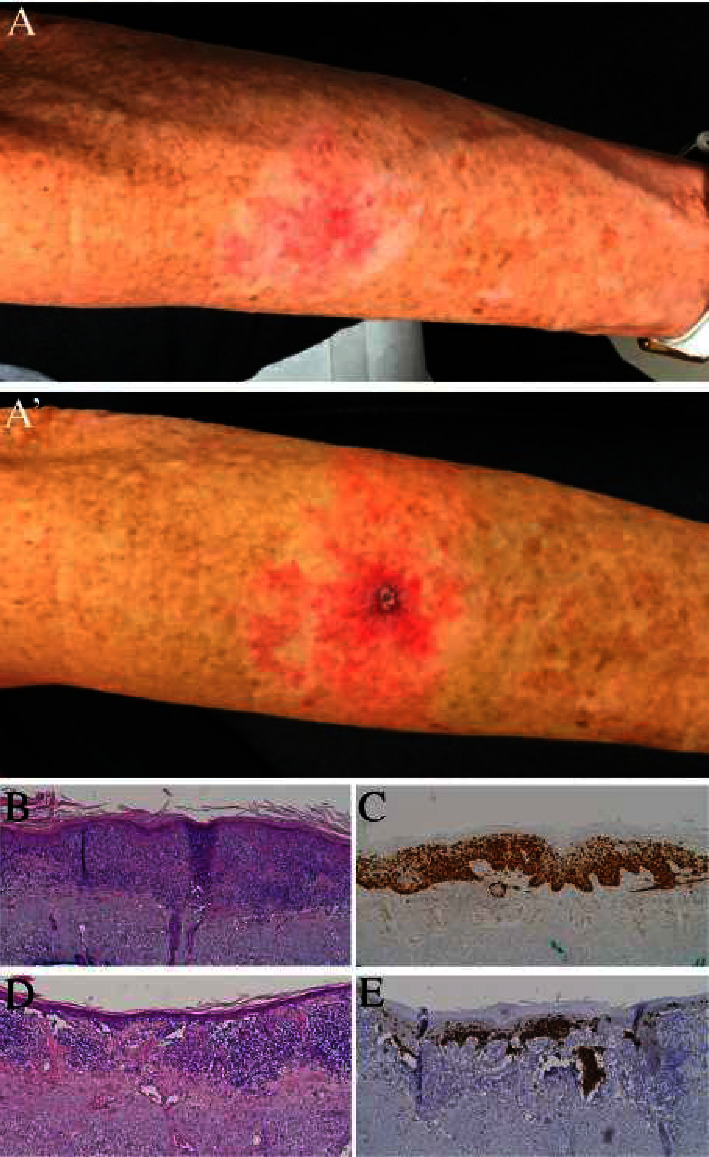
(a) A 5 cm ovoid, pink-white, minimally scaly, thin plaque on the arm of a 69-year-old female, Fitzpatrick skin type II. (a′) Postbiopsy. (b) H&E and (c) Melan-A immunohistochemistry revealing melanoma in situ, lentigo maligna type, on shave biopsy. (d) H&E and (e) Melan-A immunohistochemistry revealing invasive lentigo maligna melanoma, Breslow depth 0.3 mm. All microscopy is performed at 100x total magnification.

A 69-year-old woman presented with an asymptomatic growth on her left arm that had consistently enlarged over the previous five years. Examination revealed a 5 cm ovoid pink-white, minimally scaly, thin plaque with intact sensation and without pain or itch (Figure 1(a), a′). This growth had been diagnosed clinically as tinea corporis and nummular dermatitis but did not respond to topical antifungals or steroids, respectively. The lesion had also been treated as an actinic keratosis with cryotherapy with subsequent growth. The patient denied a history of similar rashes and was growing increasingly frustrated with the lack of treatment response.

A shave biopsy was performed with additional clinical differential diagnoses, including lichen sclerosis and Bowen's disease. Pathology was consistent with a diagnosis of melanoma in situ, lentigo maligna-type (Figures 1(b) and 1(c)). The excision of the clinical residual was significant for invasive lentigo maligna melanoma, Breslow depth 0.3 mm (Figures 1(d) and 1(e)). Per our institutional protocol [5], she was treated with off-label imiquimod prior to Mohs surgery, three times weekly for 4 months, without perceptible improvement, though given the lack of pigmentation in this neoplasm, it was noted that response was challenging to assess. She subsequently underwent slow Mohs micrographic surgery with immunologic staining for Melan-A and Sox10, without residual melanoma being identified [5–8]. The defect was repaired linearly and healed well.

## 3. Discussion

Amelanotic melanoma arises from malignant melanocytes that do not produce mature melanin granules, with variable presentations, including amelanotic papules and nodules, desmoplastic plaques, subungual neoplasia with nail plate deformity, or secondary amelanotic growths that have metastasized from a primary pigmented melanoma [9]. The risk of developing a melanoma is modifiable, with the risk of melanoma doubling after 5 sunburns and halved with regular sunscreen use [10]. The ABCDE criteria recommended for patient evaluation of melanocytic neoplasia are not designed to help patients evaluate at-risk hypopigmented lesions [10]. However, as with other skin cancers, dermatoscopy and biopsy are indicated for all suspicious hypopigmented lesions, especially if the lesion is refractory to other treatments [3]. Most patients with lentigo maligna-type melanoma have localized disease at presentation, but once this type of melanoma reaches lymph node or distant metastasis, outcomes are similar to those of other melanoma subtypes, hence the need to diagnose accurately and treat in a timely manner [11, 12]. The increased time to diagnosis and definitive surgical treatment resulting from initial misdiagnosis and treatments for infectious and inflammatory etiologies in this case may have resulted in dermal invasion and increased risk for recurrence and metastasis [13, 14].

The key clinical features of this case presentation were the asymptomatic, slow-growing nature of this neoplasm and the failure to respond to multiple different treatment modalities for nonneoplastic etiologies. Amelanotic melanoma can be diagnostically challenging. Therefore, our aim with this case report is to bring increased awareness to clinicians of the need to biopsy a “rash” that does not present with classic symptoms (i.e., itch) or respond to standard topical treatments.
